# Quality of life following two different techniques of an open ventral hernia repair for large hernias: a prospective randomized study

**DOI:** 10.1186/s12893-022-01551-w

**Published:** 2022-03-17

**Authors:** Andrija Antic, Stefan Kmezic, Vladimir Nikolic, Dejan Radenkovic, Velimir Markovic, Ilija Pejovic, Lidija Aleksic, Zlatibor Loncar, Svetlana Antic, Jelena Kovac, Ljiljana Markovic-Denic

**Affiliations:** 1 Clinical Center of Serbia, Clinic of Digestive Surgery, Belgrade, Serbia; 2 Faculty of Medicine, University of Belgrade, Belgrade, Serbia; 3 Clinical Center of Serbia, Emergency Center, Belgrade, Serbia; 4 Military Medical Academy, Belgrade, Serbia; 5 Clinical Center of Serbia, Institute of Radiology, Belgrade, Serbia

**Keywords:** Incisional hernia, Quality of life, Rives, Segregation component technique, EuraHS

## Abstract

**Background:**

We compare the health-related quality of life (QoL) of patients with incision hernias before and after surgery with two different techniques.

**Methods:**

In this prospective randomized study, the study population consisted of all patients who underwent the first surgical incisional hernias repair during the 1-year study period. Patients who met the criteria for inclusion in the study were randomized into two groups: the first group consisted of patients operated by an open Rives sublay technique, and the second group included patients operated by a segregation component technique. The change in the quality of life before and 6 months after surgery was assessed using two general (Short form of SF-36 questionnaires and European Quality of Life Questionnaire—EQ-5D-3L), and three specific hernia questionnaires (Hernia Related Quality of Life Survey-HerQles, Eura HS Quality of Life Scale—EuraHS QoL, and Carolinas Comfort Scale—CCS).

**Results:**

A total of 93 patients were included in the study. Patients operated on by the Rives technique had a better role physical score before surgery, according to the SF-36 tool, although this was not found after surgery. The postoperative QoL measured with each scale of all questionnaires was significantly better after surgery. Comparing two groups of patients after surgery, only the pain domain of the EuraHS Qol questionnaire was worse in patients operated by a segregation component technique.

**Conclusion:**

Both techniques improve the quality of life after surgery. Generic QoL questionnaires showed no difference in the quality of life compared to repair technique but specific hernia-related questionnaires showed differences.

## Background

Incisional hernias are one of the most common postoperative complications of abdominal surgery. The frequency of these hernias depends on the type of surgery performed. The incidence of incisional hernias after laparotomy varies from 3 to 20% [1, 2], while after laparoscopic surgery it is much less, about 0.8–2.8% [3, 4].

The underlying disease, emergency surgery, type and length of incision, surgical site infections, as well as age, male sex, obesity, and smoking, represent risk factors for the incision hernia [4–6].

In recent years, many techniques and new materials for the operation of incisional hernias have been developed. The component-separation technique (CST), with the use of autologous tissue and its variations, was described by Mathes and Bostwick in 1977 and Ramirez in 1990 [7–9]. The greatest drawback of this technique is the high recurrence rate, as much as 53% [10]. Sublay technique (Rives) has proven very effective, with low recurrence rates (0–23%) and minimum rates of complications. Among its disadvantages are the complexity of the surgery, the longer duration of surgery, and the likely persistence of chronic abdominal pain [11].

The postoperative outcome is traditionally assessed as survival or improvement of symptoms associated with the disease. However, these measures do not emphasize the overall perception of the patient about the impact of an operation on a subjective experience. For this reason, quality of life (QoL) measures have been developed and used to measure the effectiveness of therapeutic interventions. As QoL is greatly disturbed in patients with incisional hernias, more attention is paid to this outcome after surgery. Several factors affect the QoL, and some of the most significant are pain, mobility impairment, cosmetics, and length of convalescence [12].

This study aimed to compare the health-related quality of life of patients with incision hernias before and after surgery with two different techniques.

## Methods

### Study design, study setting and patients

In this prospective randomized study, the study population consisted of all patients who were operated at the Clinic for Digestive Surgery, Clinical Center of Serbia, during the 1-year study period. Indications for surgery were: pain, marked discomfort, and episodes of visceral incarceration. Only symptomatic patients who underwent the first surgical incisional hernias repair were included in this study, after gaining informed consent. Including criteria were: large incisional hernia (> 10 cm midline), without signs of infection, and persons older than 18 years. Excluding criteria for this study were: persons under 18 years of age, pregnancy, mentally or cognitively unable to be consented, parietal defect less than 10 cm, wound infection, previous abdominal surgery with some herniological technique (recurrent hernia), poor preoperative health status (ASA > 3—a scoring system of the American Society of Anesthesiologists), obesity (body mass index (BMI) > 25), chemotherapy, radio or corticosteroid therapy at the time of surgery, emergency hernia surgery due to inverting hernia.

Patients who met the criteria for inclusion in the study were randomized by the computerized list of numbers into the two groups: the first group consisted of patients operated by an open Rives sublay technique using a large polypropylene mesh (Rives group—RS group), and the second group included patients operated by a segregation component technique (CST group—open hernia repair). The used mesh was Parietene™ flat sheet mesh (monofilament polypropylene mesh 30 cm × 30 cm, weight 70 g/m^2^ with pore size of 1.8 mm × 2.1 mm) in all patients operated by Rives sublay technique. The mesh was fixed in the retromuscular preaponeurotic space. To avoid bias, the patient was told just immediately before surgery which type of surgery would be performed. The staff was not blinded, but to avoid the influence of a different level of surgical techniques, patients operated by only two surgeons were included in the study. Participating surgeons have already performed at least 50 open and 50 laparoscopic incisional hernia repairs. Written consent was obtained from each patient before joining the study. For each patient, anamnestic, clinical, and laboratory preoperative data were collected: age, sex, BMI, tobacco use, comorbidities, including cardiovascular diseases, diabetes, cancer, and physical status classification according to the American Society of Anesthesiologists (ASA). The duration of surgery, intraoperative and early postoperative complications, were collected, as well as the total length of hospitalization. Patients were monitored during hospitalization and 6 months after surgery. The following outcomes were observed: fascial defect (cm), other postoperative complications, and quality of life before surgery and 6 months after surgery. According to the organization of our health system, patients have a regular check-up in the hospital by their surgeon 6 months after the operation. Therefore, we preferred face-to-face interviews with participants to telephone interviews before that time.

### Questionnaires

The change in the quality of life was assessed using five questionnaires. Two general and three specific hernia questionnaires were used. Before surgery, patients completed the following questionnaires: Short form of SF-36 questionnaires, European Quality of Life Questionnaire (EQ-5D-3L), Questionnaire on quality of life with a hernia (Hernia Related Quality of Life Survey-HerQles), and European Registry of Abdominal Wall Hernias Quality of Life (Eura HS Quality of Life Scale—EuraHS QoL). Six months after the operation, besides the abovementioned questionnaires, the Carolinas Comfort Scale (CCS) was also used.

The SF-36 is a generic quality-of-life questionnaire that covers eight health domains: physical functioning (PF), role limitations due to the physical problems (RP), bodily pain (BP), general health perceptions (GH), vitality (VT), social functioning (SF), role limitation due to emotional problems (RE), and mental health (MH). These domains are scored on a scale of 0 (poor health) – 100 (best health) and can be presented in two major subscales: physical component summary (PSC) and mental component summary (MSC) [13]. We used the Serbia translation of this questionnaire.

The EQ-5D, as a measure of health conditions, comprises two parts: the EQ-5D descriptive system and the EQ-5D visual analogue scale (EQ-5DVAS). EQ-5D descriptive system measures health status by following five dimensions: mobility, self-care, usual activities, pain/discomfort, and anxiety/depression. Each level can be coded as a number 1, 2, or 3, which indicates having no problems for 1, having some problems for 2, and having extreme problems for 3. EQ-5D index was calculated using population norms for Germany, because there were not the data for our population. Zero in this index indicates ‘death’ and 1 ‘full health’. Besides, patients marked their own view of overall health on the vertical analogue scale (EQ-5D VAS) which can range from 0 (worst imaginable) to 100 (best imaginable) [14].

As a specific hernia questionnaire, the HerQles, EuraHS QoL, and CCS were used. Specific HerQles is a specific hernia-related quality of life Likert-style questionnaire that includes 12 single items. For easier interpretation, the score was transformed to a 0 to 100 scale using simple equitation 100-point scale [15]. Larger numbers on scale represent a better QOL. EuraHS QoL is a specific hernia questionnaire with 9 questions across 3 domains: pain, restriction of activities, and cosmetic discomfort. The total score ranges from 0 (best QoL) to 90 (worst QoL). CCS is a 23-item, Likert type questionnaire that measures, on a scale of 0 to 5 severity of pain, sensation, and movement limitations from the mesh in the following eight categories: laying down (LD), bending over (BO), sitting up (SU), activities of daily living (ADL), coughing or deep breathing (CB), walking (W), stairs (S), and exercise (E). The CCS score is derived by totaling the scores from each of the 23 items. The total score ranges from 0 (best QoL) to 115 (worst QoL). Patients who did not answer more than 2 questions were not included in the calculation of the total score [16]. The CCS score is mainly validated in postoperative time of administration due to the high focus on mesh sensation which makes the CCS more appropriate for use in postoperative settings [17]. It is an ideal tool for the assessment of QoL post hernia repair [18]

Our study was approved by the Ethical Committee of the Clinical Center of Serbia in accordance with the principles of the Declaration of Helsinki (2000 revision of Edinburgh). No experimental surgery; All the surgical techniques used are already part of our daily practice.

### Statistical analysis

With an α error of 0.05, a study strength of 0.80, and a ratio of the number of test subjects in the groups 1:1, it was estimated that 90 patients (45 in each group) would be required to see a change in the observed outcome (quality of life) (change in the EQ5D questionnaire from 60 to 80 points). For data descriptions, the arithmetic mean and standard deviation, median, absolute and relative frequencies were used. Checking the normalization of data distribution was done using the Kolmogorov–Smirnov test. Student's T-test was used to analyze continuous data with a normal distribution, while the Wilcoxon test of rank ranges was used for continuous data without normal distribution. For bonded samples with normal distribution, the t-test for bonded samples was used, while the Wilcoxon test was used for data that did not have a normal distribution. P values  < 0.05 were considered statistically significant. Hi square and McNemar tests were used to analyze discrete numerical data. Statistical analysis was performed using the SPSS program version 20.0 (SPSS Inc., Chicago, IL, USA).

## Results

A total of 93 patients (mean age 58.3 years, range 28–78 years; 55.9% of males,) were included in the study. The separation component technique was used in 49 patients (CST group), and Rives-Stoppa sublay technique was used in 44 patients (RS group). There are no significant differences regarding main demographic and clinical characteristics between the two groups of operated patients (Table 1).

**Table 1:** Demographic and clinical characteristics of patients

	Overall	CST group	RS group	p value (CST vs Rives)
	No (%)			
*Demographic data*				
Number of patients	93	49 (52.7)	44 (47.3)	
Gender				
Male	52 (55.9)	31 (63.3)	21 (47.7)	0.132
Female	41 (44.1)	18 (36.7)	23 (52.3)	
Age	58.3 ± 11.6	59.7 ± 9.4	56.7 ± 13.5	0.606
One or more comorbidities	29 (62.4)	29 (59.2)	29 (65.9)	0.504
Comorbidities				
Congestive heart failure	17 (18.3)	7 (14.3)	10 (22.7)	0.293
Peripheral vascular disease	15 (16.1)	9 (18.4)	6 (13.6)	0.536
Diabetes mellitus type 2	19 (20.4)	9 (18.4)	10 (22.7)	0.603
Cancer	23 (24.8)	9 (18.3)	14 (31.8)	0.149
BMI (kg/m^2^)	26.4 ± 5.9	25.1 ± 2.9	27.5 ± 7.4	0.733
ASA score				
1	7 (7.5)	2 (5.7%)	5 (12.8)	0.651
2	46 (49.5)	24 (68.6)	22 (56.4)	
3	21 (22.6)	9 (25.8)	12 (30.8)	
*Intraoperative data*				
Hernia size (cm^2^)	11.7 ± 8.4	11.8 ± 8.4	11.6 ± 8.6	0.940
Duration of surgery (min)	137.5 ± 61.0	141.1 ± 59.5	133.6 ± 63.1	0.565
*Postoperative data*				
Length of stay (days)	17.4 ± 27.2	22.1 ± 36.2	12.1 ± 8	0.125
Blood transfusion	16 (17.2)	6 (12.2)	10 (22.7)	0.181
Subcutaneous drainage	64 (68.8)	33 (67.3)	31 (70.5)	0.747
*Early wound complication*				
Seroma	1 (1.1)	1 (2.0)	0 (0)	0.341
Hematoma	2 (2.2)	2 (4.1)	0 (0)	0.175
Wound.infections	6 (6.5)	4 (8.2)	2 (4.5)	0.478

*ASA* American Society of Anesthesiologists, *BMI* body mass index, *CST* compenent separation technique, *RS* Rives-Stoppa

The results of the functional measures and QoL assessment preoperatively and 6-months after surgery for all patients using generic and specific hernia-related questionnaires are presented in Table 2. The postoperative means of each scale of the SF-36 questionnaire were significantly higher for seven domains than those recorded preoperatively: RF (µpreop = 34.5; µpostop = 78; p < 0.001), RP (µpreop = 17.3; µpostop = 78.3; p < 0.001), BP (µpreop = 39.7; µpostop = 80.7; p < 0.001), GH (µpreop = 41.9; µpostop = 66.2; p < 0.001), as well as, both summary scores: PCS (µpreop = 35.7; µpostop = 75.7; p < 0.001), and MCS (µpreop = 43.3; µpostop = 75.7; p < 0.001). Only mental health showed the non-significant decrease.

**Table 2:** Preoperative and 6-months functional postoperative outcome measure and QoL assessment scale

Questionnaire		Before surgery (mean ± SD)	Total filed questionnaires n (%)	6-months after surgery (mean ± SD)	Total filed questionnaires n (%)	*p* value
*Generic health questionnaires*						
*SF-36* (0–100)			91 (97.8)		91 (97.8)	
Physical functioning	PF	34.5 ± 25.7		78.0 ± 24.5		< 0.001
Role physical	RP	17.3 ± 28.8		78.3 ± 34.2		< 0.001
Body pain	BP	39.7 ± 24.2		80.7 ± 24.1		< 0.001
General health	GH	49.1 ± 19.3		66.2 ± 21.3		< 0.001
Vitality	VT	51.5 ± 10.0		57.8 ± 14.2		< 0.001
Social functioning	SF	44.5 ± 21.6		76.1 ± 22.9		< 0.001
Role emotional	RE	22.0 ± 36.2		81.7 ± 35.2		< 0.001
Mental health	MH	54.2 ± 11.1		50.9 ± 11.1		0.021
Physical component summary	PCS	35.7 ± 18.9		75.7 ± 22.1		< 0.001
Mental component summary	MCS	43.3 ± 13.6		66.6 ± 16.2		< 0.001
*Health—EuroQoL*						
EQ-5D index (0–1)		0.60 ± 0.32	92 (98.9)	0.94 ± 0.15	89 (95.7)	< 0.001
EQ-5D VAS Score (0–100)		41.5 ± 20.5	85 (91.4)	72.4 ± 20.3	83 (89.2)	< 0.001
*Specific hernia-related QoL questionnaires*						
*HRQLes* (0–100)		50.4 ± 13.0	92 (98.9)	73.5 ± 9.3	89 (95.7)	< 0.001
*EuraHS Qol total*		49.9 ± 20.9	92 (98.9)	16.2 ± 15.5	92 (98.9)	< 0.001
EuraHS Qol—pain		12.2 ± 7.6		2.8 ± 4.3		< 0.001
EuraHS Qol—restriction of activities		21.9 ± 11.4		7.6 ± 8.7		< 0.001
EuraHS Qol—cosmetic discomfort		15.9 ± 4.9		5.7 ± 4.9		< 0.001

*EQ-5D* European Quality of Life Questionnaire, *VAS* visual analogue scale, *HerQles* hernia related quality of life survey, *EuraHS QoL* European Registry of Abdominal Wall Hernias Quality of Life

A greater increase was observed in the physical component summary which included physical functioning (PF) and role physical (RP) with the greatest increase. The role emotional (RE) showed the greatest increase in mental component summary.

The mean pre-operative EQ-5D index improved from 0.60 to 0.94 (p < 0.001), and the mean EQ-VAS from 41.5 to 72.4 (p < 0.001).

Using a specific hernia-related QoL HRQLes questionnaire, patients showed significantly better QoL after surgery (Table 2). Also, EuraHS Qol total score and each specific domain after surgery showed significantly better Qol (Table 2).

Analyzing the difference in the quality of life concerning the operative technique before the operation, it was noticed that the CST group and the RS group differ only in the role physical domain of the SF-36 questionnaire. Patients operated by the Rives technique had a better RP preoperative score (26.2 vs 10.9, p = 0.023) but the physical component summary did not differ between the two groups. Other domains of the SF-36 questionnaire as well as EQ-5D-3L, VAS scale, HRQoL, EuraHS QoL questionnaires did not show a significant difference before surgery compared to the operative technique. Although before surgery the CST group and the RS group differed in the RP scale, after surgery, this difference was absent (87.2 vs. 79.2, p = 0.345). Comparing these two groups of patients after surgery, a difference was observed only in the pain domain of the EuraHS Qol questionnaire, which was worse in patients operated by a segregation component technique. Table 3 shows the quality of life, before and after surgery, assessed using generic and specific hernia-related questionnaires according to operative techniques.

**Table 3:** Preoperative and 6-months functional postoperative outcome measure and QoL assessment scale, according to operative techniques (CST group and RS group)

Questionnaire		Before surgery				After surgery			
		CST group (mean ± SD)	RS group (mean ± SD)	Total filed n (%)	p	CST group (mean ± SD)	RS group (mean ± SD)	Total filed n (%)	p
*Generic health questionnaire*									
*SF-36* (0–100)				91 (97.8)					91 (97.8)
Physical functioning	PF	33.1 ± 23.5	36.8 ± 28.2		0.264	81.2 ± 19.5	79.4 ± 24.9		0.968
Role physical	RP	10.9 ± 23.0	26.2 ± 35.0		**0.023**	87.2 ± 25.7	79.2 ± 34.1		0.345
Bodily pain	BP	39.6 ± 21.8	40.9 ± 28.0		0.220	83.2 ± 19.7	82.1 ± 24.9		0.936
General health	GH	48.7 ± 20.3	48.8 ± 18.0		0.841	66.9 ± 17.0	68.2 ± 21.5		0.554
Vitality	VT	50.5 ± 11.1	52.5 ± 8.9		0.642	59.4 ± 10.5	56.9 ± 16.3		0.886
Social functioning	SF	43.0 ± 18.6	46.3 ± 24.9		0.849	76.8 ± 21.4	77.6 ± 22.5		0.531
Role emotional	RE	18.7 ± 32.9	27.1 ± 41.3		0.608	93.5 ± 20.0	79.6 ± 35.9		0.159
Mental health	MH	54.6 ± 8.5	53.8 ± 6.1		0.611	51.6 ± 6.4	50.3 ± 13.2		0.976
Physical component summary	PCS	33.1 ± 17.3	38.5 ± 20.3		0.176	76.7 ± 20.0	74.7 ± 24.4		0.654
Mental component summary	MCS	41.7 ± 12.2	44.9 ± 15.0		0.257	67.3 ± 15.2	65.7 ± 17.5		0.635
*Health—EuroQoL*									
EQ-5D-3L index (0–1)		0.62 ± 0.30	0.58 ± 0.34	92 (98.9)	0.577	0.93 ± 0.18	0.95 ± 0.11	89 (95.7)	0.541
VAS		40.4 ± 20.0	42.9 ± 21.6	85 (91.4)	0.544	72.3 ± 17.1	72.3 ± 23.9	83 (89.2)	0.756
*Specific hernia-related QoL questionnaire*									
*HRQoL* (0–100)		50.6 ± 12.9	51.4 ± 14.9	92 (98.9)	0.783	73.3 ± 9.1	73.6 ± 9.5	89 (95.7)	0.870
*EuraHS Qol total*		49.2 ± 21.1	50.7 ± 20.9	92 (98.9)	0.719	15.3 ± 12.6	17.1 ± 18.2	92 (98.9)	0.584
EuraHS Qol—pain		11.3 ± 7.4	13.0 ± 7.9		0.286	1.9 ± 2.6	3.8 ± 5.4		**0.030**
EuraHS Qol—restriction of activities		21.9 ± 11.6	21.9 ± 11.2		0.983	7.5 ± 8.0	7.7 ± 9.6		0.931
EuraHS Qol – cosmetic discomfort		15.9 ± 5.2	15.8 ± 4.6		0.940	5.9 ± 4.8	5.6 ± 5.0		0.766

*CST* compenent separation technique, *RS* Rives-Stoppa, *EQ-5D* European Quality of Life Questionnaire, *VAS* visual analogue scale, *HerQles* hernia related quality of life survey, *EuraHS QoL* European Registry of Abdominal Wall Hernias Quality of Life

Analysis of postoperative CCS symptoms scores is presented in Table 4. Overall QoL scores were 5.35 ± 6.89 in the CST group and 8.70 ± 13.68 in the Rives group (p = 0.159). Statistical differences between these two groups were only observed for mesh sensation, Rives group showed a higher mean of this domain. Pain and movement scores did not differ in these two observed groups.

**Table 4:** Mean Carolinas Comfort Scale Scores of two patients groups

*Carolinas comfort scale* (CCS) Total filed questionnaires 83 (89.2%)	CST group (mean ± SD)	Rives group (mean ± SD)	P value
Sensation score	1.55 ± 2.61	3.56 ± 4.81	**0.019**
Pain score	1.97 ± 2.53	2.57 ± 4.69	0.463
Movement score	1.84 ± 2.83	2.56 ± 4.73	0.396
Carolinas score total	5.35 ± 6.88	8.70 ± 13.68	0.159

*CST* Component separation technique RS–Rives-Stoppa

## Discussion

Our study showed that quality of life after elective surgery for incisional hernia was significantly better than preoperative status regardless of which surgical technique was used.

We used two generic health questionnaires, SF-36 Short Form Health Survey and EQ-5D. As prior studies showed that a disease-specific questionnaire is more useful than a general questionnaire in the evaluation of the changes in quality of life, we also used two specific hernia-related QoL questionnaires, HRQLes and EuraHS QoL, and one scale that measures the patient satisfaction following hernia repair. To the best of our knowledge, this paper is one of the few in which several QoL questionnaires have been applied [19], especially to compare the quality of life after the application of two different surgical techniques.

When we compare the preoperative and postoperative quality of life using the SF-36 questionnaire, it may be noted that all daily activities were significantly improved after hernia repair, excluding mental health. The role of incisional hernia surgery in improving the quality of life mostly can be observed in the physical subscales of the SF-36 instrument, additionally summarized in the PCS. Although the preoperative physical activity was worse in patients who underwent the CST technique, postoperative improvement in this domain was observed in both groups, without significant differences between them. Only item "mental health" showed a non-significant decrease after the operation. Nevertheless, the overall MCS score showed a significant improvement in postoperative function in both study groups, and our respondents indicated improvement in general health. It seems that physical, social functioning, and emotional aspect had a greater impact on participants´ well-being in both groups, which were observed in other studies [20]. Moreover, we observed an increase in the VAS scale from 41.5 to 72.4 on a scale of a maximum of 100, regardless of applied surgical technique. This score is a widely used health assessment method [21] because it is sensitive and easy to use. The improvement in general health also was revealed by the EQ-5D tool. In the prospective, multicenter, COBRA study, conducted in 9 centers in the USA and the Netherlands [22], it was found the significant improvement in health at 6 months onto 24-months postoperatively compared to preoperative baseline values at EQ-5D index and EQ-5D VAS scale.

HerQLes questionnaire, as an abdominal wall hernia-specific tool, allows patients to score their own physical and emotional status. The main advantage of this questionnaire is the ability to compare the quality of life after incisional hernia repair with different techniques and especially to compare techniques that use prosthetic mesh vs. those without mesh [15]. In our study, this tool showed that the quality of life 6 months after surgery was significantly better, regardless of which of the two surgical techniques was applied.

In line with previous scores, the EuraHS Qol questionnaire also showed improvement after surgery in the overall score and in all three elemental components of quality of life (pain, restriction of activities, and cosmetic discomfort) in our study, which already had been proved as the most important health assessment after hernia repair [16]. Preoperative pain is considered a risk factor for postoperative chronic pain [5]. In our study, the CST and Rives-Stoppa groups did not differ in the pain domain nor the other EuraHS Qol questionnaire domains before surgery. However, six months after hernia repair, patients who underwent surgery by the Rives-Stoppa technique had a worse pain domain in the EuraHS Qol questionnaire. Although the Rives-Stoppa sublay technique is very popular, with low recurrence rates and minimal complications, the main disadvantage is the possibility of chronic abdominal pain, which explains the poorer values of the pain domain in this group of patients [23]. The specific questionnaire for hernia repair could find the differences in two types of surgery as it was the case regarding pain in the Rives-Stoppa group, which was not possible with a generic questionnaire.

The CCS is a widely accepted QoL questionnaire that is primarily used to assess QoL after hernia repair and validated in all hernia types undergoing mesh repair [24]. Also, perhaps most importantly, it is well accepted by patients. It is shown that patients prefer the CCS by a 3 to 1 ratio over the SF-36 survey because of its specificity and ease to use [25]. We found that at 6 months point after hernia repair there was no statistically significant difference in pain and movement score as well as in Carolinas total score between CST and Rives-Stoppa group. However, the mesh sensation score was worse in patients with Rives-Stoppa hernia repair. The sensation score for component separation was at the same level as in a study conducted by Klima et al. [26], while Forester et al. Reported a slightly higher sensation score for open incisional hernia repair with mesh than it was revealed in our study [27]. The difference between these two groups in sensation may be due to the difference in the technique of these two types of operations. Namely, the component separation technique is based on subcutaneous lateral dissection, fasciotomy lateral to the rectus abdominis muscle, and dissection on the plane between external and internal oblique muscles with medial advancement of the block that includes the rectus muscle and its fascia [10]. What is essential is that the tissue used to cover the defect remains innervated, which is not the case with the Rives-Stoppa technique with sublay mesh position where lack of innervated tissue may lead to increased mesh sensation [26].

It is suggested that both uses of generic and hernia-specific QoL questionnaires represent the gold standard [24]. It is important to emphasize the difference in marking pain in CCS and EuraHS-Qol, that is the observed statistical difference among two groups in EuraHS related to pain which was not the case in the analysis of pain according to CCS. The possible explanation can be the differently formulated issues in these two questionnaires. The CCS has special questions about sensation and pain, while in EuroHS-Qol, there are only questions about the pain. In an additional analysis of dichotomous data, we found that 73.1% of patients in the RS group that marked “sensation of mesh” in the CCS, marked “pain” in EuraHS-Qol. Further studies about the sensitivity of pain detection with a higher number of patients operated by RS technique using more different questionnaires are necessary. However, we already found that specific hernia-related questionnaires have shown greater sensitivity in detecting differences between patients operated with different techniques.

The limitation of this study was the assessment only 6-months after surgery. Most studies that used different HoL tools revealed an initial drop in the quality of life one month after surgery and an increase in further periods [22]. Second, we did not conduct a total blind study. However, the type of surgery was explained to the patients immediately before the operation. The advantage of this study was the fact that the patients were operated on by two surgeons with similar experiences. This avoids selection bias, ie the operation of less complex patients by a less experienced surgeon. Besides, all patients with Rives-Stoppa hernia repair had the same mesh features which avoid the influence of different mesh features on patient pain, sensation, and quality of life. Fourth, although it was shown in the literature reviews that complete assessment of the quality of life after hernia surgery is best achieved using a general scale with a specific scale and a validated pain scale [28], we did not use a specific pain score. We assessed pain using items in the SF-36 questionnaire, EuraHS QoL questionnaire, and CCS.

## Conclusion

Overall, both techniques lead to improved quality of life after surgery. Generic QoL questionnaires showed no difference in the quality of life compared to repair technique but specific hernia-related questionnaires showed some differences in pain domain EuraHS Qol questionnaire and sensation score CCS.

## Data Availability

The datasets used and/or analysed during the current study are available from the corresponding author on reasonable request.

## Abbreviations

CST: Component-separation technique
QoL: Quality of life
ASA: A scoring system of the American Society of Anesthesiologists
BMI: Body mass index
RS group: Rives group
EQ-5D-3L: European Quality of Life Questionnaire
HerQles: Hernia Related Quality of Life Survey
EuraHS QoL: European Registry of Abdominal Wall Hernias Quality of Life
CCS: Carolinas Comfort Scale
PF: Physical functioning
RP: Role limitations due to the physical problems
BP: Bodily pain
GH: General health
VT: Vitality
SF: Social functioning
RE: Role limitation due to emotional problems
MH: Mental health
PSC: Physical component summary
MSC: Mental component summary
LD: Laying down
BO: Bending over
SU: Sitting up
ADL: Activities of daily living
CB: Coughing or deep breathing
W: Walking
S: Stairs
E: Exercise
VAS: Visual analogue scale
